# COQTEL project dataset : Corrosion quantification trough extended use of Lamb waves

**DOI:** 10.1016/j.dib.2025.111393

**Published:** 2025-02-13

**Authors:** C. Nicard, M. Rébillat, O. Devos, M. El May, F. Letellier, S. Dubent, M. Thomachot, M. Fournier, P. Masse, N. Mechbal

**Affiliations:** a PIMM^1^1 https://x.com/LabPimm/., CNAM / CNRS / Arts et Métiers Institute of Technology, Paris, France; b I2M^2^2 https://x.com/LaboI2M/., University of Bordeaux / CNRS / Arts et Métiers Institute of technology, Bordeaux, France; c Cedrat Technologies, Meylan, France; d RESCOLL, Talence, France

**Keywords:** Piezoelectric transducers, Sparse array, Pitting corrosion, Structural health monitoring, Ultrasonic Lamb waves, Controlled electrochemical experiments

## Abstract

Corrosion poses significant safety and cost challenges in the aeronautic industry. Ultrasonic Lamb Waves (LW), emitted and received by a sparse array of piezoelectric elements (PZT), offer an efficient, cost-effective, and versatile solution for corrosion monitoring. This dataset corresponds to two experiments involving a LW solution based on a sparse PZT array and able to monitor corrosion pit growth on a 316L stainless steel plate during controlled corrosion. The corrosion pit size is electrochemically controlled by the imposed electrical potential and the injection of a corrosive NaCl solution through a capillary at the desired pit location. Simultaneously, the corrosion pit growth is monitored in-situ every 10 seconds using a sparse array of 4 PZTs bonded to the back of the steel plate. Two independent experiments were conducted to assess the repeatability of this approach. The collected dataset collected can facilitate the development of Structural Health Monitoring (SHM) algorithms and methodologies, provide data for waves/damage interaction modeling, and help bridging the gap between research and industry in this domain.

Specifications Table

**Table:** 

Subject	Electrochemistry
Specific subject area	A single corrosion pit is formed on a steel plate while in parallel Lamb waves are regularly sent and received in the plate using four bonded piezoelectric elements
Type of data	Raw
Data collection	To perform corrosion experiments, a standard three electrodes setup connected to a VersaSTAT 3F potentiostat is used and potential and current are recorded. During corrosion, Lamb waves are emitted and received by 4 bonded piezoelectric transducers (PZT) managed by a Generation Unit and a Lamb Waves Detection System from Cedrat Technologies and a National Instrument acquisition board (NI USB-6366). A custom MATLAB script configures one PZT in actuator mode at a central frequency of 200 kHz and the three others in sensing mode. One measurement is available roughly every 10 seconds and precise time stamps are stored.
Data source location	Data have been collected at I2M, University of Bordeaux / CNRS / Arts et Métiers Institute of technology, Bordeaux, France and are stored at PIMM, CNAM / CNRS / Arts et Métiers Institute of Technology, Paris, France.
*Data accessibility*	*Repository name: ZENODO* *Data identification number:*10.5281/zenodo.14193335 *Direct URL to data:*https://zenodo.org/records/14193336
*Related research article*	*C. Nicard, M. Rébillat, O. Devos, M. El May, F. Letellier, S. Dubent, M. Thomachot, M. Fournier, P. Masse & N. Mechbal, “In-situ monitoring of µm-sized electrochemically generated corrosion pits using Lamb Waves managed by a sparse array of piezoelectric transducers”, Ultrasonics, Elsevier, In press (Accepted)*

## 1 Value of the Data

• A localized pitting corrosion experiment is instrumented with piezoelectric elements able to send and receive ultrasonic Lamb waves
• This database allow to assess in-situ the effect of actual corrosion pit damages on Lamb waves along with electrochemical parameters (potential and current)
• This dataset can be reused by research teams working on structural health monitoring (SHM) of aeronautic structures and on ultrasonics waves modelling
• These data can help in developing new SHM algorithms related with damage monitoring and prognosis or to benchmark already published ones
• These data can help to validate numerical models of the interaction between Lamb waves and corrosion damage by providing actual data with increasing damage sizes

## 2 Background

The presented dataset corresponds to the article ``In-situ monitoring of µm-sized electrochemically generated corrosion pits using Lamb waves managed by a sparse array of piezoelectric transducers'' [1] published in Open Access in Ultrasonics (Elsevier) journal and is available in Open Access online [2] Monitoring in real time and autonomously the health state of structures is referred to as Structural Health Monitoring (SHM [3,4] Ultrasonic technologies relying on Lamb Waves (LW) are extremely sensitive to corrosion damage and can be easily automated thanks to bonded piezoelectric elements (PZTs) [5,6] LW inspection methods based on PZTs have however never been validated on corrosion pits realistic of actual corrosion (from 10 µm to 150 µm) but rather on large generalized corrosion areas (in cm) using dens [[6], [7], [8], [9]] or spars [[10], [11], [12]] PZTs arrays. This is because it was yet not possible to generate a single corrosion pit in a controlled manner and to simultaneously and in-situ record LW interacting with such a damage. The generation of single corrosion pits using micro capillaries can be performed where proper condition (concentrations and potential) allowing to create a single pit are met [13] This method has been adapted to suit the simultaneous presence of PZTs managing LW emission and reception. The objective of this dataset is thus to provide the scientific community with experimental data corresponding to an electrochemically controlled growth of a µm-sized corrosion pit simultaneously monitored it in situ by means of LW emitted and received using PZTs.

## 3 Data Description

The COQTEL data available online on the repository [2] are organized within two HDF5 format files corresponding to each independent experiment and named “*Essai_Corrosion1.h5*” and “*Essai_Corrosion2.h5*”. HDF5 files are useful data containers that can be organized internally as *Groups* with some *Attributes* that corresponds to the metadata associated with each Group and that can contain either other *Groups* or *Data*. The HDF5 file format has been chosen here as it is an Open Source Format that can be read by many scientific computing languages (such as Matlab, Python) and for which many information and example codes are publicly made available (see for example [14].

Within one of the two HDF5 file provided in the dataset, an attribute called *“PZT_info_geo”* provides the geometrical positions (*x,y*) of the 4 PZTs bonded on the plate. Then, in each HDF5 files, the data corresponding to one experiment are organized as follows:

- The electrochemical data (time [min], potential [V] and current [A]) are within a Group called “*EC_data*”. These data are time series with a total of *N* samples and corresponds to electrochemical measurements achieved at the moments where Lamb Waves have been acquired. No specific attributes are attached to the “*EC_data*” group.
- Each other Group denoted as “*State_n*”(where n is an integer ranging between 1 and *N*) corresponds to a given Lamb waves data at a given corrosion pit size corresponding to the time stamp *n* within the “*EC_data*” group. No specific attributes are attached to the “*State_n*” groups.
- Within each Group “*State_n*”, a group denoted “*200kHz_5cycles*” is dedicated to specify the burst test signal that has been used (here a 5 cycles tone burst at 200 kHz). Attributes attached to this group are the sampling frequency (2 MHz), the central frequency (200 kHz), the number of cycles (5 cycles) and the name of the signal (Burst 200kHz 5 cycles).
- Within each burst test signal group, one group is attributed to each actuator. This group is named “*ActionneurK*” with *K* the actuator number ranging between 1 and 4 as 4 PZTs are available in the present experiment. No specific attributes are attached to the “*ActionneurK*” groups. These groups contain data that correspond to a matrix denoted “*measured_data_rep_1.mat*” with one dimension corresponding to discrete time and the other dimension organized as follows: time value, actuation signal, and measured signals. For example, if the emitting transducer is the PZT number 3, this second dimension is organized as: time value, signal sent to PZT 3, and signals measured by PZTs 1, 2, and 4.

**Fig. 1:**
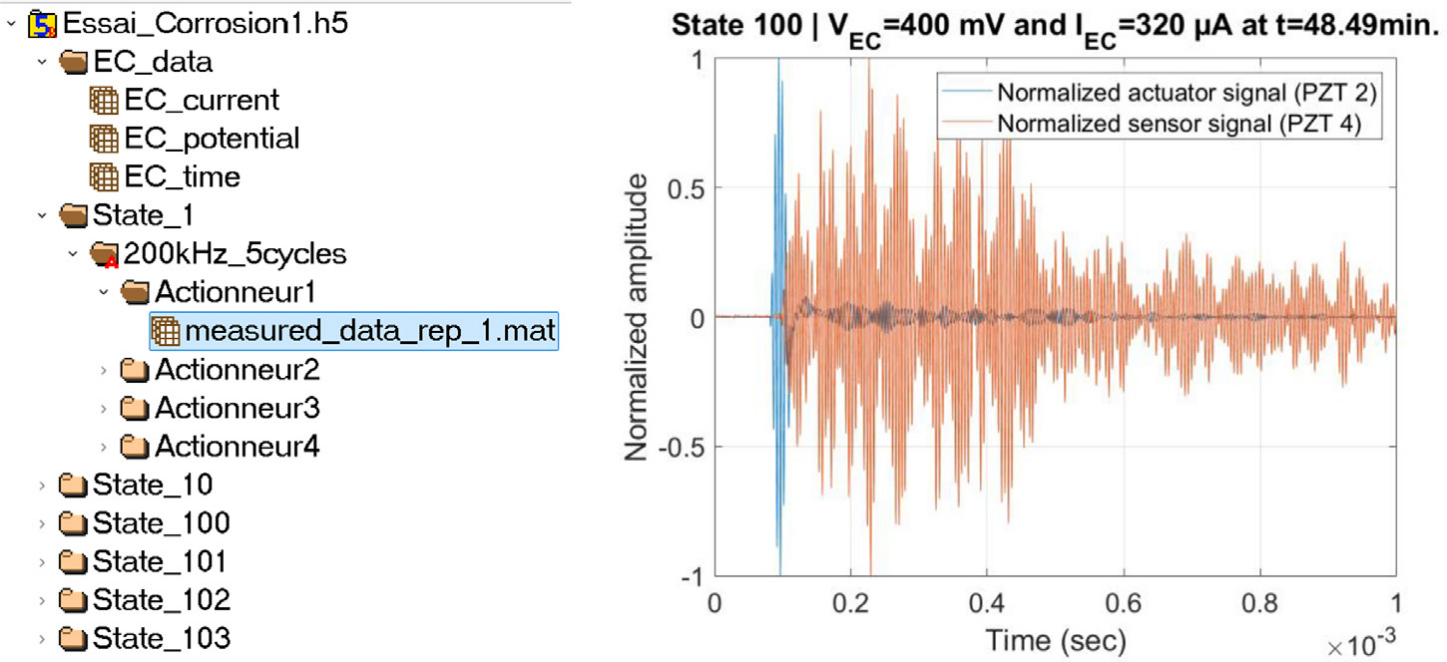
[Left] HDF5 file organization of one experiment. [Right] Snapshot of data contained within the database.

An overview of the *“Essai_Corrosion1.h5”* file using *HDFView* software is shown in Fig. 1 [Left]. The various Groups detailed previously along with the corresponding data can be seen on that Figure.

An example Matlab code allowing to read the data is also provided along with the two datasets. It demonstrates how to load data for a given corrosion state, a given PZT actuator and a given PZT sensor. An example of such extracted data is shown in Fig. 1 [Right]. As the measured signal is of lower amplitude than the exciting one, both time series have been normalized in amplitude.

## 4 Experimental Design, Materials and Methods

The experimental protocol presented here is the same one that allowed to collect the data that are at the basis of the research article [1] Consequently, the texts and images presented in this section are largely reproduced from the experimental protocol already published in [1].

### 4.1 Experimental samples

**Fig. 2:**
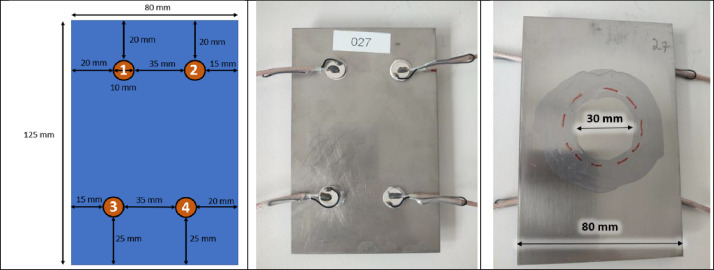
Experimental steel sample under study. [Left] Position of the PZTs composing the sparse network. [Middle] Back face of the sample with the 4 bonded PZTs. [Right] Front face of the sample partially varnished before applying the corrosion cell (reproduced from [1]).

The experimental samples to be monitored are 1.5 mm thick 316L stainless steel plates measuring 80 mm by 125 mm. Four PZT transducers (Noliac NCE51, Ø10 mm × 1 mm) are bonded to these plates at specific geometric positions (Fig. 2 [Left]), avoiding symmetries and staying away from the edges to limit Lamb wave (LW) reflections. A 150 mm coaxial cable is brazed onto each element to optimize the signal-to-noise ratio and enable LW transmission and reception. The PZTs are glued following Cedrat Technologies' bonding procedure which is as follows: i) layer with abrasive paper the surface on which the patch will be glued, ii) clean the sanded surface with ethanol, iii) place a drop of Loctite EA 9492 glue on the sanded part, iv) place the patch over the glue and press to spread the glue over the surface of the patch, v) coarsely clean the glue that spills over the sides, vi) let the glue dry at room temperature for 24 hours. Two similar samples have been manufactured. The front face of the samples (which will be corroded) was hand-polished with SiC grinding paper up to 1200 grade and then cleaned with ethanol to prevent premature corrosion. To avoid under-joint corrosion, the area around the contact between the sample and the corrosion cell is predetermined and varnished with an Acrylic Protective Lacquer (Electrolube), leaving a ≃30 mm non-varnished disc at the center where corrosion pit damage will be generated.

### 4.2 Electrochemical experimental setup

**Fig. 3:**
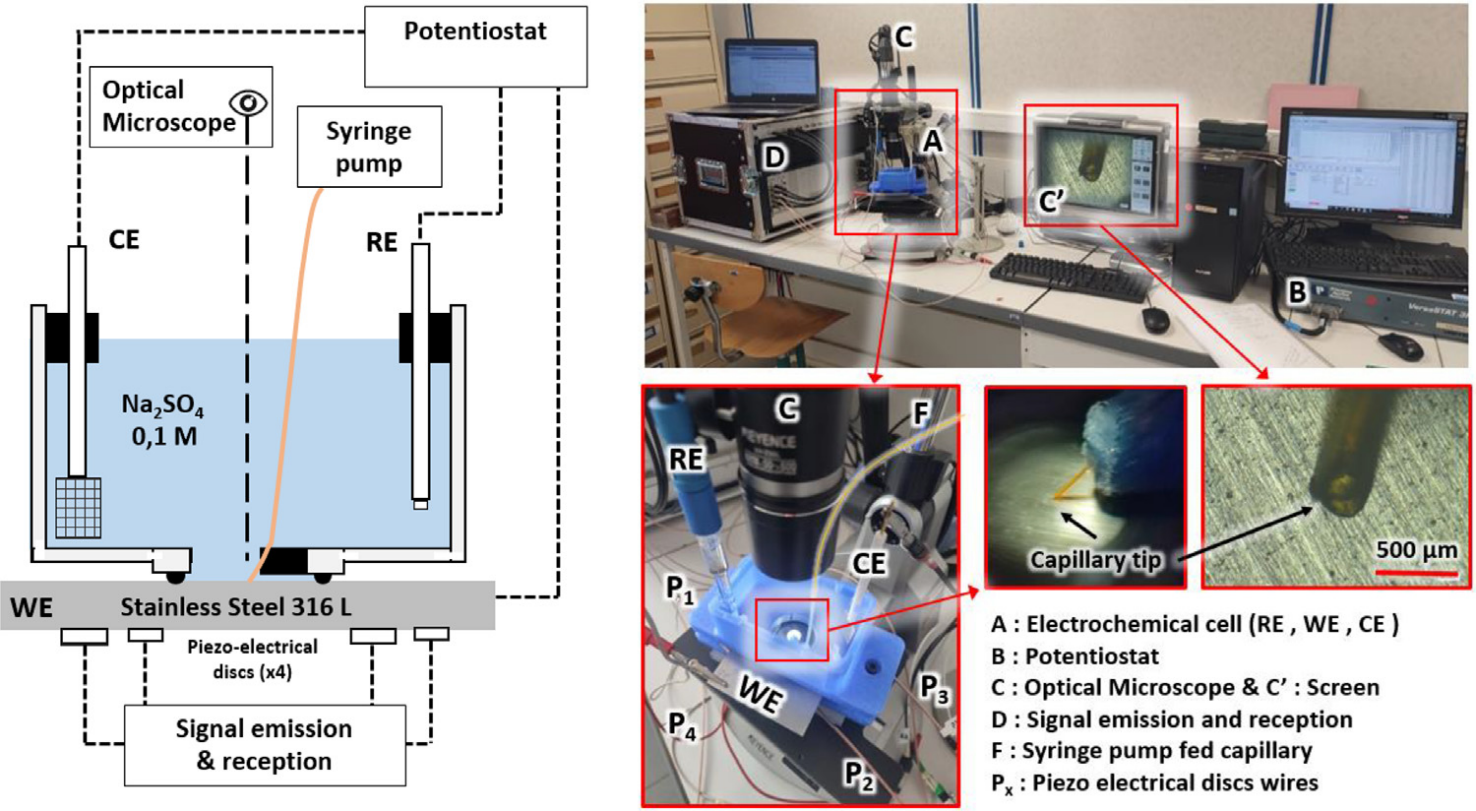
[Left] Schematic of experimental set up and [Right] Pictures of experimental setup with zooms on the specific equipment's details in the text (reproduced from [1]).

A dedicated electrochemical cell (Fig. 3 [Left]) was 3D printed using PETG filament, measuring 96 mm × 60 mm × 48 mm and holding approximately 150 mL of electrolyte. It features a capillary guide for injecting corrosive species like chloride ions at a 30° angle to the steel surface, connected to a syringe pump for controlled injection. The design allows direct optical microscopic observation using a VH-Z50L long focal objective (Keyence). Fig. 3 [Right] shows the experimental setup. For corrosion experiments, a standard three-electrode setup is used: the Working Electrode (WE) is the 316L stainless steel sample, the Counter Electrode (CE) is a 20 mm × 20 mm platinum grid, and the Reference Electrode (RE) is a silver chloride electrode (Ag/AgCl). The CE and RE are immersed from the top, while the steel sample (WE) is pressed against the cell's bottom with an O-ring to ensure water tightness and prevent PZT stress. The electrodes are connected to a VersaSTAT 3F (Ametek) potentiostat. This setup aims to generate localized corrosion near the capillary tip and control the dissolution rate to create a pit with a controlled volume. Two relevant potentials were identified: P1 = 150 mV vs Ag/AgCl (passive domain) and P2 = 400 mV vs Ag/AgCl (dissolution domain) for stainless steel in both NaCl concentrations.

### 4.3 Controlled generation of localized corrosion damage

#### 4.3.1 Experiment 1

**Fig. 4:**
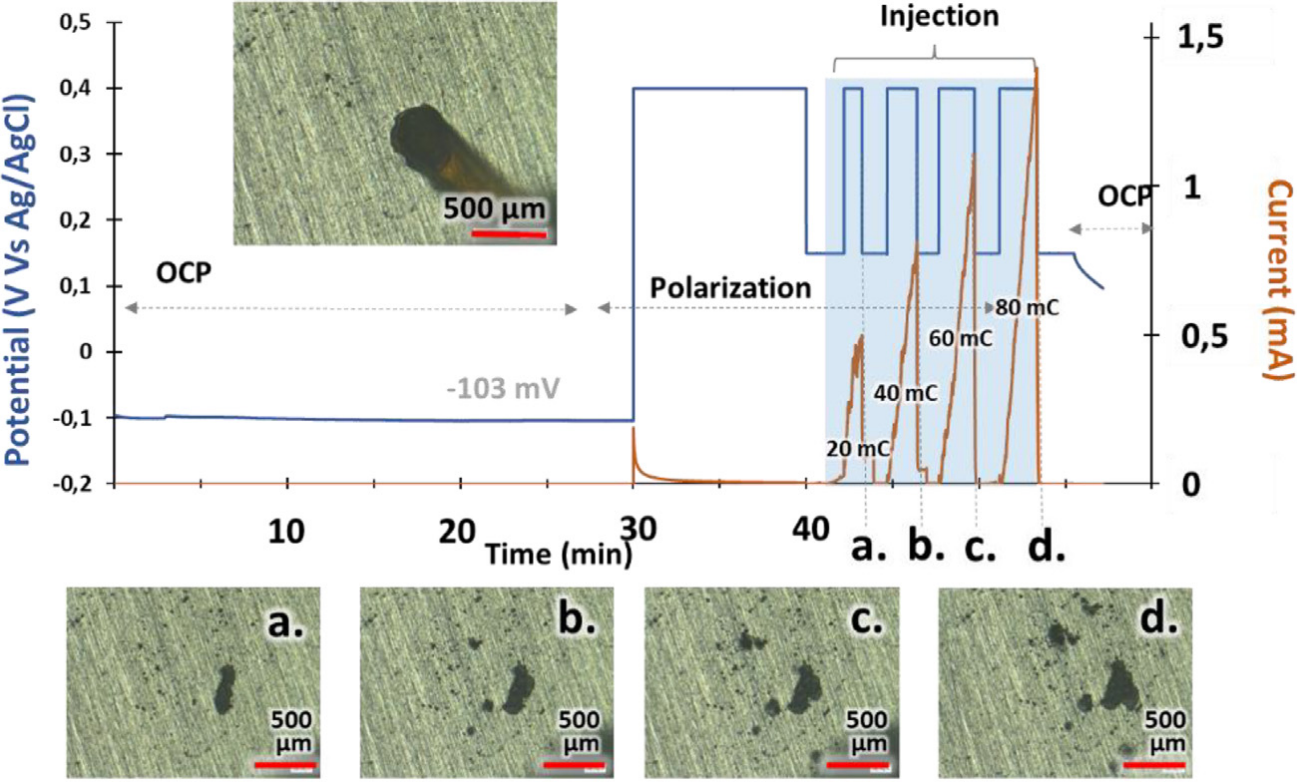
Experiment #1. Measured potential and current versus time. t_i_: initial time of injection. (a, b, c, d): pictures from optical microscope (reproduced from [1].

• **Setup:** Electrochemical cell with Na2SO4 0.1M solution (S1). Capillary (170 µm Ø) connected to syringe pump with Na2SO4 0.1M + NaCl 4M solution (S2).
• **Procedure:**
  1. Measured Open Circuit Potential (OCP) for 30 min to ensure passive behavior.
  2. Applied potential P2 for 10 min without chloride injection; material remained passive.
  3. Applied potential P1 without chloride injection; material stayed passive.
  4. Injected S2 solution at 0.1 µL/s. Current increased, indicating localized dissolution.
  5. Alternated between P2 (pit growth) and P1 (stop dissolution) based on cumulative charge criteria (20, 60, 120, 200 mC).
  6. Photographs taken during P1 to monitor damage size.
• **Results**: Fig. 4 shows electrochemical potential vs. time and current measurements.

#### 4.3.2 Experiment 2

**Fig. 5:**
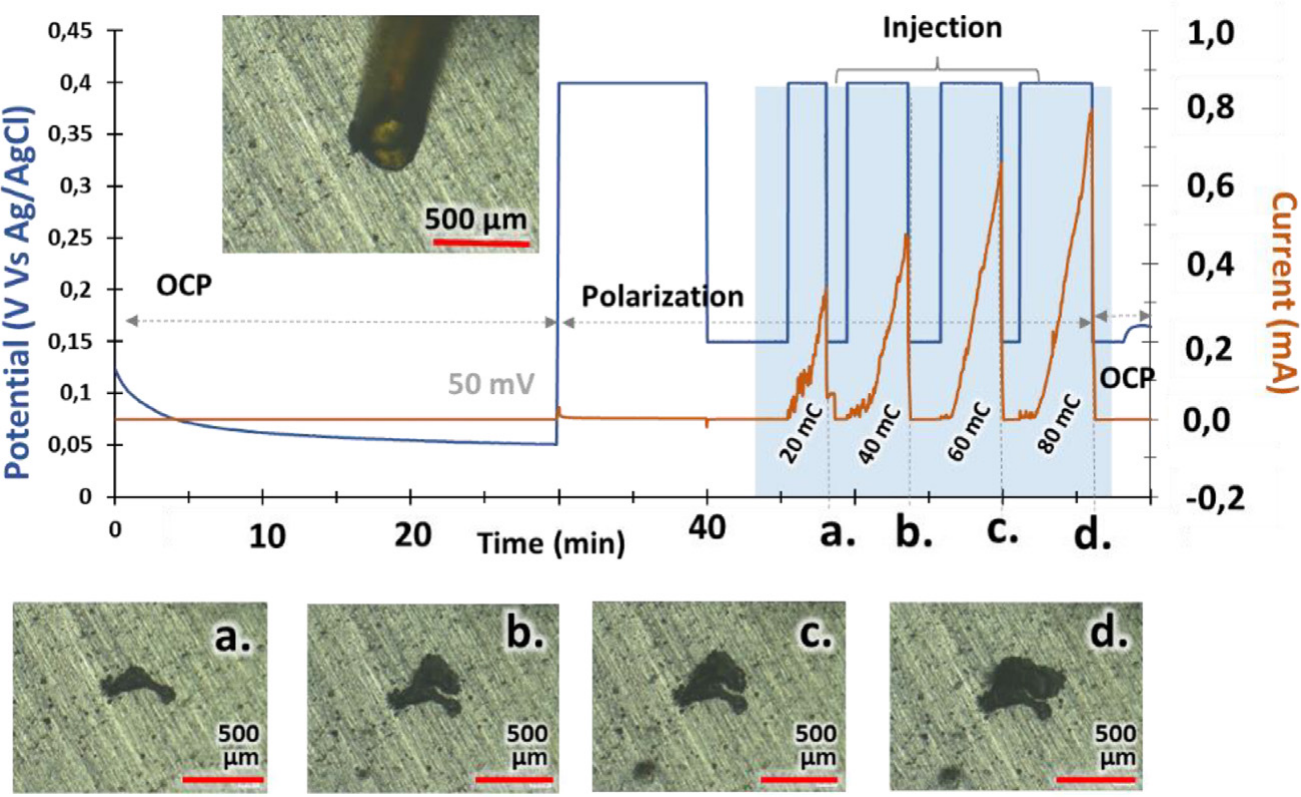
Experiment #2. Measured potential and current versus time. t_i_: initial time of injection. (a, b, c, d): pictures from optical microscope (reproduced from [1]).

• **Setup:** NaCl concentration decreased to 3M, capillary diameter increased to 220 µm.
• **Procedure:** Similar to experiment 1
• **Results:**Fig. 5 shows similar results to Experiment 1, confirming protocol repeatability.

### 4.4 In-situ ultrasonic Lamb waves measurements

**Fig. 6:**
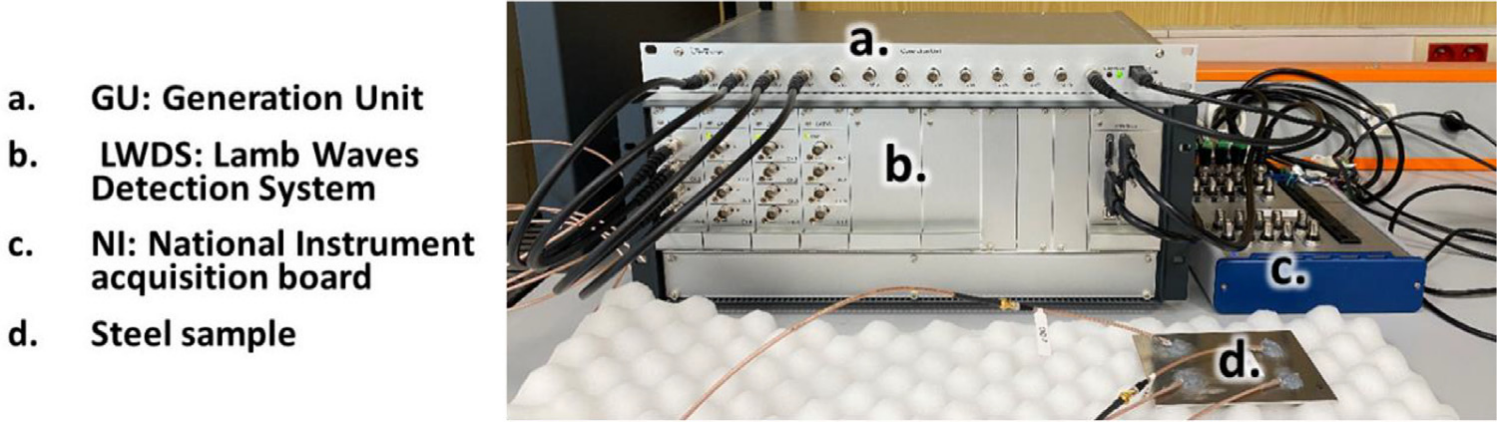
Overview of Cedrat Technologies LW management system (reproduced from [1]).

During the corrosion experiments, Lamb waves (LW) are emitted and received by a sparse array of 4 PZTs bonded to the back of the steel sample (Fig. 2 [Middle]). The LW management system, provided by Cedrat Technologies and denoted as D in Fig. 3 [Right], is detailed in Fig. 6. It comprises three blocks:

1. **Generation Unit (GU):** Generates signals for up to 12 channels.
2. **Lamb Waves Detection System (LWDS):** Contains up to 16 channels, each with a power amplifier (±15V, 1A) and a switching system for toggling between emitting and receiving modes.
3. **National Instrument (NI) Acquisition Board (NI USB-6366):** Acquires signals at a 2 MHz sampling frequency.

Only 4 channels of the GU, LWDS, and NI systems are used. A MATLAB script configures one PZT in actuator mode (pulse mode) and the other three in sensing mode (echo mode) in a round-robin fashion. The PZT in pulse mode generates a Lamb wave at 200 kHz, which is received by the other PZTs and measured by the NI interface. The time to collect (∼50 ms) and store (∼9.5 s) signals for the 4 PZTs is approximately 10 seconds. Measurements are taken continuously during corrosion and precise time stamps are stored along with each measurement.

## Limitations

**Fig. 7:**
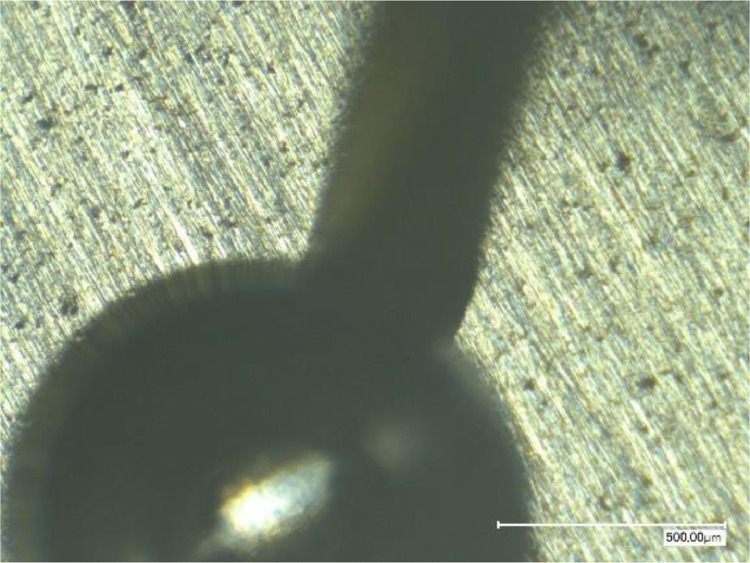
Picture of the bubble appeared during the second experiment (reproduced from [1]).

This experimental protocol relies on a very thin capillary which drives the flow of corrosive liquid to the desired pit location. Unexpectedly, during the second experiment, a bubble having a radius of **≃500** µm appeared at the output of the capillary as some air has been accidentally trapped into it (see Fig. 7). This bubble has been manually evacuated a few minutes after its apparition. In the dataset “*Essai_Corrosion2*”, the bubble appeared at State 30 to and has been removed at State 100.

## Data Availability

ZenodoCOQTEL dataset: Corrosion Quantification Through Extended use of Lamb waves (Original data).
